# A Behaviourally Anchored Checklist for Mental Health Occupational Therapy Intake Interviews: Development and Reliability in a Single-Station Standardised Patient Encounter

**DOI:** 10.5334/pme.2026

**Published:** 2026-05-07

**Authors:** Yasuhisa Nakamura, Shohei Mori, Shuji Kijima, Masahiro Tanaka, Osamu Taguchi

**Affiliations:** 1 Department of Rehabilitation, Division of Occupational Therapy, Faculty of Health Sciences, Nihon Fukushi University, Handa City, Aichi Prefecture, Japan; 2 Faculty of Rehabilitation, School of Health Sciences, Fujita Health University, Toyoake City, Aichi Prefecture, Japan

## Abstract

**Introduction:**

Intake interview performance is a core competency in mental health occupational therapy education, but structured and reliable assessment tools for brief observed encounters remain limited. This cross-sectional study therefore developed a 16-item behaviourally anchored checklist/rubric for intake interviews and examined scoring reliability in a single OSCE-format standardised-patient station.

**Methods:**

Sixty third-year occupational therapy students from two Japanese universities completed one station using one of three standardised-patient scenarios. Two licensed occupational therapists independently rated performance across three domains (attitude, interview skills, evaluation). Internal consistency was assessed using Cronbach’s alpha, inter-rater agreement using intraclass correlation coefficients (ICCs; absolute-agreement definition), and Bland–Altman analysis was used to quantify rater agreement and assess fixed or proportional bias in total scores.

**Results:**

Scenario-specific inter-rater agreement for the total score ranged from 0.761 to 0.929 across the three scenarios. Domain-level ICCs ranged from 0.584 to 0.892 for attitude, 0.510 to 0.825 for interview skills, and 0.794 to 0.979 for evaluation. Pooled descriptive summaries showed high internal consistency for all domains (Cronbach’s α = 0.850–0.887) and for total scores (α = 0.816–0.817). Bland–Altman analysis showed a small mean difference between raters (0.18) and 95% limits of agreement from –2.35 to 2.71.

**Discussion:**

This behaviourally anchored checklist/rubric showed high internal consistency and acceptable inter-rater agreement for scoring intake interview performance in a single-station context. By operationalising interpersonal competencies as observable behaviours, the instrument may support rater calibration and structured formative feedback, including in settings where multi-station OSCE examinations are not feasible.

## Introduction

Objective Structured Clinical Examinations (OSCEs) are the gold standard for assessing clinical competencies in health professions education (HPE), particularly in medical and nursing programmes [1,2]. As described by Harden, the OSCE is a structured approach that samples performance in standardised clinical tasks and applies structured scoring instruments, often with simulated or standardised patients (SPs) [3]. While conventionally implemented as multi-station circuits [4,5], some programmes use a single “OSCE-format station” in routine teaching. In this manuscript, we define an “instrument” as the behaviourally anchored checklist/rubric used for scoring within such a station.

In mental health occupational therapy education, a core assessment target is performance during an intake interview—the initial clinical information gathering and brief clinical observation of a client. Conceptually, this performance integrates (i) an interpersonal stance that supports engagement (e.g., empathic communication and appropriate nonverbal behaviour), (ii) professional conduct that maintains safety and boundaries, and (iii) brief clinical observation and synthesis while responding to clients’ narratives in real time [6,7]. Written or knowledge-based examinations capture this construct poorly because performance is context-dependent and unfolds through interaction. If assessment relies mainly on written tests, it risks overemphasising theoretical knowledge while underrepresenting practical and interpersonal competencies central to psychiatric care [8]. Educators, therefore, must judge not only what learners know but also how they enact skills under the uncertainty of a live interview.

While behavioural checklists for interview performance exist across multiple health disciplines, a systematic review of OSCE communication-skill checklists reported substantial heterogeneity in checklist design and in the reporting and strength of reliability and validity evidence [9]. This literature indicates that observable criteria can structure scoring, but it also suggests two persistent gaps. First, many tools provide limited transparency about how the construct was defined and translated into items and domains, making adaptation and rater training difficult. Second, reliability and validity evidence is often uneven or incompletely reported for time-limited interview stations. Moreover, in mental health occupational therapy education, intake interview performance is often judged using global or subjective impressions [10,11], potentially reducing transparency and weakening scoring consistency across raters and cohorts, particularly with multiple instructors.

To address this gap, we developed a behaviourally anchored checklist/rubric to support structured scoring of intake interview performance in mental health occupational therapy education. We adopted an educational assessment perspective that emphasises behavioural anchoring, that is, translating complex competencies into observable indicators with explicit performance-level descriptors. This approach is suitable for time-limited observed interviews, in which raters must judge performance under uncertainty, because behavioural anchors can reduce ambiguity and support behaviour-focused feedback [12,13,14].

This study aimed to develop a behaviourally anchored checklist/rubric for scoring performance in a simulated clinical intake interview delivered as a single OSCE-format station in mental health occupational therapy education and to examine the reliability of instrument scoring, focusing on internal consistency and agreement between raters.

## Materials and Methods

### Study design and setting

This cross-sectional observational study was conducted between September 2024 and July 2025 at two Japanese universities offering accredited occupational therapy programmes. At both universities, station scores contributed to the module grade; however, the present study used these data only to evaluate scoring reliability (internal consistency and inter-rater agreement) and did not assess educational outcomes.

Each student completed one OSCE-format station only. This single-station format was used to embed the assessment within routine teaching under practical constraints, including cohort size, limited rater availability, room capacity, and SP resources. A conventional multi-station OSCE would have required parallel rooms, additional SPs and raters, and substantially more assessment time. Accordingly, the present study was designed to generate station-level evidence on scoring reliability rather than exam-level dependability across multiple stations.

Each station consisted of a 5-minute intake interview with an SP, followed by a 5-minute scoring period during which raters completed the 16-item checklist/rubric. Three scenarios were used, and students were randomly assigned so that each scenario included 20 students overall (10 per scenario at each university).

### Participants

#### Participant groups and roles

This study involved four distinct groups. (1) A pilot cohort of 20 second-year occupational therapy students was used in 2023 to test feasibility and refine station prompts, the student-SP script/training guidance, and the behavioural anchors; pilot data were not included in the psychometric analyses. (2) The main study cohort consisted of 60 third-year undergraduate students (30 per university) who completed one OSCE-format SP intake interview station; their scores were analysed for reliability. (3) Student standardised patients (student SPs), who did not belong to the assessed cohorts, portrayed the cases during the stations. (4) Two licensed occupational therapists served as raters and independently scored all 60 third-year students using the final checklist/rubric.

#### Main study participants

Sixty third-year undergraduate occupational therapy students participated (30 from each university). At both universities, the station was scheduled after students had completed core mental health occupational therapy coursework and as preparation for upcoming clinical placements, where initial client interviews are expected under supervision. Accordingly, the station targeted foundational intake-interview behaviours appropriate for third-year learners (e.g., professional stance, structured questioning/listening, and brief observation/synthesis) rather than advanced diagnostic formulation or treatment planning. The station assessment was compulsory as a course requirement; however, the use of assessment data for research required written informed consent. Students who were absent or did not complete the full station were excluded.

### Ethics

The study was approved by the Ethics Committee of Nihon Fukushi University (Approval No. 23-042-02), and written informed consent was obtained from all participants.

### Checklist/Rubric Development

The behaviourally anchored checklist/rubric was developed in four steps. First, we defined the target construct as performance during an initial mental health intake interview, including professional/interpersonal conduct, interview technique, and assessment-related reasoning, and we specified an initial construct map. This construct map integrated three literature streams: occupational therapy literature on therapeutic communication and professional/relational stance in mental health practice [6,7,15], evidence from OSCE-format and simulation-based assessment on checklist/rating design and the use of behaviourally anchored descriptors to support reliable scoring [12,13,16], and psychiatric interview literature emphasising the elicitation, observation, and brief synthesis of clinically relevant signs and symptoms during initial assessment [17]. The resulting construct map and its operationalisation in the station context are summarised in Supplementary Table S4.

Second, we specified three domains—attitude, interview skills, and evaluation—and drafted candidate items and behaviourally anchored 0–2 scoring descriptors suitable for real-time station-based scoring, using the construct map, commonly taught intake interview behaviours in the participating programmes, and relevant literature [6,7,12,13,15,17]. Third, the research team iteratively reviewed and revised item wording and anchor descriptions through expert-panel discussions involving occupational therapy educators specialising in mental health (panel characteristics are summarised in Supplementary Table S2). Across rounds, the panel reviewed each item for relevance to the construct map, clarity, redundancy, domain allocation, and anchor specificity for real-time scoring, using the literature-informed domain definitions as decision rules [6,7,12,13,15,17]. Items were revised, merged, or deleted until consensus was reached on a feasible set of observable behaviours aligned with the construct map.

Fourth, we piloted the draft instrument and refined it based on feasibility feedback. To improve readability, details of pilot testing and refinement are provided in Supplementary Appendix 1. To make the evidence-to-design chain explicit and facilitate replication and local adaptation, we added an item-to-construct-and-literature mapping table (Supplementary Table S3), which links each item to the construct map and supporting literature. We did not conduct a formal Delphi process or content validity index procedure during this initial development phase; more formal content validation remains a task for future work.

### Domain framework and item allocation

We organised items into three domains—attitude, interview skills, and evaluation—to operationalise the construct map for structured scoring in a single OSCE-format station and to support rater calibration and behaviour-focused feedback [12,13,16,18]. Domain definitions were used during expert-panel review to allocate and refine items, and served as literature-informed decision rules to minimise construct overlap across domains [6,7,15,17]. “Attitude” captures professional and relational stance observable through verbal and nonverbal communication [6,7,15]. “Interview skills” captures observable interview-process behaviours that support interview flow and information gathering; items such as seating and distance were assigned to this domain because they shape engagement and interview flow in this station context [19]. “Evaluation” captures structured observation and brief synthesis/reporting of the client’s presentation, supporting early clinical reasoning in mental health contexts [17]. Items were allocated using these operational definitions and refined until consensus was reached. The final checklist/rubric comprised 16 items: attitude (4), interview skills (9), and evaluation (3).

In this manuscript, “domain score” refers to the sum of item scores within a domain for this single OSCE-format station. We use “domain score” rather than “scale” to avoid implying a subscale intended for aggregation across multiple stations, which cannot be examined in a single-station design [20,21].

### Pilot testing and refinement

A pilot test was conducted in 2023 with a separate cohort of 20 second-year occupational therapy students from one participating university to identify feasibility problems in the station workflow and unclear wording in the behavioural anchors. Based on feedback from assessed students and student SPs, we refined the interview prompts, clarified behavioural anchors, and adjusted the SP script and training guidance; details are provided in Supplementary Appendix 1.

### Procedure

Each student completed one standardised patient (SP) intake interview delivered as a single OSCE-format station. The station represented a simulated initial clinical assessment interview rather than an interview for programme admission or placement selection. The station required an introductory, supervised-level interview and brief observation/synthesis; it did not require diagnostic decision-making or treatment planning. After the station, students received structured formative feedback based on the checklist/rubric as part of routine teaching; however, feedback outcomes were not analysed in the present study. Students were informed that one of three scenarios would be presented at random. We randomly assigned students to scenarios using a random number table and capped assignments at 10 students per scenario per university to balance distributions across sites. This resulted in 20 students per scenario overall (10 per scenario at each university; total N = 60). Because each student completed only one scenario, scenario-level comparisons reflect between-student differences.

### Standardised patient training

Standardised patients were undergraduate students who did not belong to the target cohort. Under faculty supervision, SP training included reviewing scenario-specific video examples, rehearsing roles through mock interviews, and receiving feedback to ensure consistency in symptom presentation and interaction style across sessions. “Mock interviews” refers to rehearsal role-plays for SP standardisation and was not part of the study assessment data. This approach is consistent with recent studies supporting the reliability and authenticity of student SPs [22].

### Rater scoring and independence

Each interview was observed and independently scored by two licensed occupational therapists with more than 10 years of clinical experience in mental health; the two raters scored all 60 students. Blinding was not implemented because raters were aware of each student’s university of origin and assigned scenario. Interviews were video recorded, and recordings were available for review when needed to confirm real-time judgments. Raters used separate scoring sheets, were seated in the same room, and were instructed not to communicate about ratings during the interview or scoring period. Any discussion was limited to scheduled calibration meetings and did not involve reviewing or changing individual scores. Raters scored all 16 items in real time using predefined behavioural anchors.

### Rater training and calibration

Before data collection, raters completed standardised training including familiarisation with the checklist/rubric, joint review of pilot station videos, and consensus discussions to align interpretations of each behavioural anchor [16]. Brief calibration meetings were held during the study period to minimise rater drift and maintain scoring consistency.

### Statistical analysis

Because each student completed only one scenario-specific station variant, the study design did not permit estimation of exam-level reliability across multiple stations, such as a full generalizability study. We therefore analysed reliability at the level of each scenario-specific station variant and report pooled estimates across all students only as supplementary descriptive summaries.

We calculated descriptive statistics (means, standard deviations, medians, and ranges) for domain scores (attitude, interview skills, and evaluation) and total scores for each rater.

Internal consistency was examined using Cronbach’s alpha, calculated separately by rater for each domain and the total score. We interpreted alpha values using commonly cited guidance (e.g., α ≥ 0.70 acceptable and α ≥ 0.80 good) [23]. These analyses were used to describe the internal consistency of the scoring instrument within this station context, rather than to estimate exam-level OSCE reliability.

Inter-rater agreement was examined using intraclass correlation coefficients (ICCs) based on a two-way random-effects model with absolute agreement. We report single-measure ICCs (ICC(2,1)) as the primary index because ratings were assigned independently by two raters, and 95% confidence intervals were calculated for all ICC estimates. We calculated ICCs for each domain and the total score within each scenario-specific station variant (Table 1). Pooled ICCs across all students are reported only as supplementary descriptive summaries (Supplementary Table S5). We interpreted ICC values using published recommendations (poor < 0.50, moderate 0.50–0.74, good 0.75–0.89, and excellent ≥ 0.90) [24].

**Table 1:** Scenario-level reliability estimates (ICC) for the single-station OSCE-format interview assessment.


SCENARIO	DOMAIN	RATER	MEAN (SD)	MEDIAN (RANGE)	SD%max	ICC (95% CI)

1 (n = 20)	Attitude (0–8)	A	7.65 (0.59)	8 (6–8)	7.4%	0.892 (0.750–0.956)

		B	7.55 (0.76)	8 (5–8)	9.5%	

1	Skills (0–18)	A	16.25 (1.45)	16.5 (11–18)	8.1%	0.510 (0.095–0.773)

		B	16.45 (1.57)	17 (14–18)	8.7%	

1	Evaluation (0–6)	A	5.10 (1.25)	6 (2–6)	20.8%	0.857 (0.657–0.942)

		B	4.80 (1.36)	5 (2–6)	22.7%	

1	Total (0–32)	A	29.00 (2.43)	30 (24–32)	7.6%	0.761 (0.487–0.898)

		B	28.85 (2.39)	29 (22–32)	7.5%	

2 (n = 20)	Attitude (0–8)	A	7.80 (0.41)	8 (7–8)	5.1%	0.584 (0.199–0.812)

		B	7.75 (0.44)	8 (7–8)	5.5%	

2	Skills (0–18)	A	16.60 (1.43)	17 (13–18)	7.9%	0.825 (0.609–0.927)

		B	16.65 (1.31)	17 (14–18)	7.3%	

2	Evaluation (0–6)	A	5.30 (1.26)	6 (2–6)	21.0%	0.794 (0.510–0.910)

		B	4.90 (1.29)	5 (2–6)	21.5%	

2	Total (0–32)	A	29.70 (2.00)	30 (25–32)	7.6%	0.855 (0.670–0.940)

		B	29.30 (2.20)	29.5 (25–32)	7.5%	

3 (n = 20)	Attitude (0–8)	A	7.65 (0.67)	8 (6–8)	8.4%	0.691 (0.376–0.864)

		B	7.75 (0.44)	8 (7–8)	5.5%	

3	Skills (0–18)	A	16.45 (1.05)	16 (15–18)	5.8%	0.719(0.415–0.879)

		B	16.50 (1.19)	17 (14–18)	6.6%	

3	Evaluation (0–6)	A	5.05 (1.85)	6 (0–6)	30.8%	0.979 (0.944–0.992)

		B	4.90 (1.89)	6 (0–6)	31.5%	

3	Total (0–32)	A	29.15 (2.56)	30 (24–32)	8.0%	0.929 (0.828–0.971)

		B	29.15 (2.73)	29 (23–32)	8.5%	


Abbreviations: ICC, intraclass correlation coefficient; CI, confidence interval; SD, standard deviation. SD%max was calculated as SD divided by the maximum possible score for the domain and expressed as a percentage. ICCs were calculated using a two-way **random-effects** model with absolute agreement; single-measure ICCs are reported. ICC reflects agreement between Rater A and Rater B and is therefore shown once per domain.

To further assess agreement between raters for the total scores, we conducted Bland–Altman analysis. We calculated the mean difference and standard deviation (SD) of the differences and derived 95% limits of agreement (LOA) as mean difference ± 1.96 × SD. We tested fixed bias using a one-sample t-test of the mean difference against zero (df = 59). We examined proportional bias using simple linear regression with the mean total score as the independent variable and the difference between raters’ scores as the dependent variable.

All analyses were conducted using IBM SPSS Statistics for Windows, Version 21.0, with a significance level of p < 0.05.

## Results

### Descriptive statistics and inter-rater reliability

Because students completed one of three scenario-specific station variants rather than rotating across multiple stations, we first report scenario-stratified descriptive statistics and inter-rater agreement (Table 1). Pooled results across all students are presented only as supplementary descriptive summaries (Supplementary Table S5).

Across the three scenarios, scores were generally high across domains for both raters, with medians near the upper bounds for attitude, interview skills, and the total score (Table 1). Dispersion (SD%max) was comparatively larger for evaluation than for attitude and interview skills, indicating greater heterogeneity in evaluation scores across scenarios (Table 1).

Inter-rater agreement varied by scenario. For the total score, ICC (2,1) ranged from 0.761 to 0.929 across scenarios (Table 1). Domain-level ICCs ranged from 0.584 to 0.892 for attitude, 0.510 to 0.825 for interview skills, and 0.794 to 0.979 for evaluation (Table 1). Confidence intervals were wider in scenario-specific analyses because each scenario included 20 students.

Pooled descriptive statistics, pooled ICCs, and internal consistency indices (Cronbach’s alpha) are provided in Supplementary Table S5 as descriptive summaries and should not be interpreted as exam-level reliability estimates.

### Inter-rater agreement and bias

**Figure 1:**
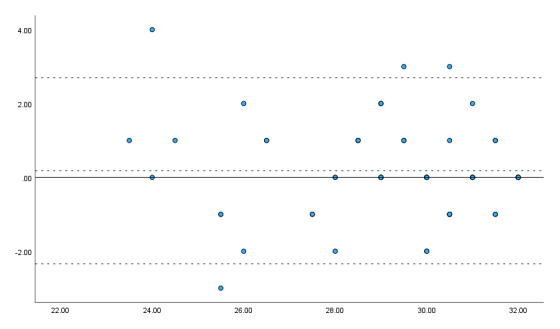
Bland–Altman plot of inter-rater agreement for total checklist/rubric scores (n = 60). *Note*. The solid line indicates the mean difference between raters (0.18), and the dashed lines indicate the 95% limits of agreement (–2.35 to 2.71). X-axis: mean of the two raters’ total scores; Y-axis: score difference (Rater A – Rater B).

A Bland–Altman analysis was used to examine agreement between the two raters’ total checklist/rubric scores pooled across scenarios (Figure 1). The mean difference (Rater A – Rater B) was 0.18 (SD = 1.29), with 95% limits of agreement from –2.35 to 2.71, indicating generally small rater differences in total-score judgments.

A one-sample t-test showed that the mean difference did not differ significantly from zero (t(59) = 1.096, p = 0.277), providing no evidence of fixed bias (Cohen’s d = 0.14). Linear regression analysis also showed no evidence of proportional bias (β = –0.045, p = 0.540). Scenario-specific Bland–Altman plots for the total score are provided in Supplementary Figures S1–S3 as exploratory analyses.

## Discussion

This study developed a behaviourally anchored checklist/rubric for scoring intake interview performance in mental health occupational therapy education and provided initial evidence on scoring reliability when the instrument was applied in a single OSCE-format standardised patient (SP) station. Because the assessment was implemented as a single station, the findings support station-level inferences about scoring reliability and rater agreement rather than exam-level dependability across multiple stations. Within this scope, the instrument showed high internal consistency, moderate-to-excellent inter-rater agreement across domains, and good agreement for the total score, suggesting that the scoring framework may support reproducible judgments in brief observed intake interview encounters.

The observed pattern is consistent with coherent domain scoring and stable total-score judgments in this station context. High internal consistency across domains suggests that items within each domain captured related facets of intake interview performance. The Bland–Altman analysis further supports stable total-score judgments by showing small mean differences between raters and no evidence of fixed or proportional bias. Together, these findings indicate that the instrument can support consistent scoring for this specific station design, while leaving open questions about generalisability to other stations, programmes, and rater groups.

Variation in inter-rater agreement across domains provides a practical signal for refinement and rater training. In the scenario-specific analyses, relatively lower agreement for interview skills was observed in Scenario 1, whereas this pattern was not consistent across scenarios. This suggests that some performance judgments may be more sensitive to scenario characteristics and item interpretation than others [15,16]. Two practical implications follow. First, behavioural anchors may need further refinement for items that require greater inference, in order to reduce interpretive latitude without changing the construct being assessed. Second, rater calibration may be strengthened through shared video exemplars and periodic checks for rater drift, which could improve reproducibility in routine teaching contexts [16]. These actions are feasible within station-based assessments and directly target the scoring process.

A key contribution of this study is the instrument design as a transferable approach to scoring complex interview performance in brief observed encounters. Behavioural anchoring translates abstract competencies, such as empathic responsiveness, professionalism, and nonverbal communication, into observable actions with explicit criteria, thereby reducing ambiguity and supporting a shared evaluative language for both assessment and feedback [12,13]. This is particularly relevant in time-limited stations, where raters must make rapid judgments and where transparent criteria can increase the specificity of feedback. To support replication and local adaptation, we provide the construct map underpinning the domain structure (Supplementary Table S4) and the item-to-construct-and-literature mapping that documents the evidence-to-design chain (Supplementary Table S3).

A distinctive feature of the instrument is its explicit evaluation domain, which requires structured observation and brief synthesis/reporting of the client’s presentation. Psychiatric intake interviews require clinicians to integrate elicited narrative information with observed presentation features, such as appearance, posture, speech, and thought-related characteristics, when forming an initial understanding of the client [17]. By making this observation-and-synthesis step explicit in the scoring framework, the instrument may increase transparency for learners and support feedback that aligns with early clinical reasoning in mental health practice. The dispersion in evaluation scores suggests that this domain may capture meaningful between-student differences that are not well represented by narrower communication checklists.

This work also addresses a broader HPE need beyond the local setting. In programmes that use interview-based SP encounters under practical constraints, a behaviourally anchored instrument can provide feasible structure for rater calibration, scoring consistency, and feedback. Transfer to other disciplines would require local calibration of anchors and, where needed, modification of items to reflect local learning outcomes and station demands [12,13].

Future research should extend the validity argument and strengthen the inferences supported by the instrument. Studies should document and test content alignment with curricular objectives, examine rater response processes and training effects [16], evaluate internal structure, and test relations to external measures of communication competence and clinical reasoning in occupational therapy education and related HPE contexts [10]. Educational consequences should also be assessed to clarify how the instrument influences learning and decision-making [10,14]. Finally, multi-station implementations should be evaluated using appropriate designs, such as Generalizability Theory, to estimate exam-level dependability and to examine the stability of domain scores when aggregated across stations.

## Limitations

This study has several limitations. First, the sample comprised third-year students from two Japanese institutions, which may limit generalisability. Future studies should include additional institutions, learner levels, and regions.

Second, the assessment consisted of a single OSCE-format station rather than a multi-station OSCE examination. Accordingly, the findings should be interpreted as evidence for station-level scoring reliability and inter-rater agreement, not exam-level dependability. In addition, the design did not allow evaluation of whether domain scores remain stable when aggregated across stations, as required for exam-level domain inferences [20,21]. Future multi-station studies should therefore use appropriate designs, such as Generalizability Theory, to examine broader dependability.

Third, although the three scenarios reflected common psychiatric presentations, they did not capture the full range of mental health conditions and settings. Because each student completed only one scenario, scenario effects were examined between students, and scenario-stratified estimates remained exploratory because of the smaller subgroup sizes. Broader scenario sets and repeated encounters or multi-station designs are therefore needed.

Fourth, although we used a staged development process with iterative review and pilot refinement, we did not undertake a formal Delphi process or other structured content-validation procedure during instrument construction. Further work should examine content representation and content validation more formally across broader educational settings.

Fifth, scores were generally high and clustered near the upper end of the rating scale, suggesting ceiling effects and restricted range. This pattern may have affected reliability estimates and limited score discrimination. Future studies should test whether revised anchors, more challenging scenarios, or samples with wider performance variability improve score spread.

Sixth, the standardised patients were undergraduate students rather than trained actors or real patients, which may have reduced realism. In addition, raters were not masked to institution or scenario and were seated in the same room, so expectancy effects and residual contamination cannot be fully excluded. Independent video-based scoring and masking of site information would strengthen rater independence where feasible.

Finally, this initial evaluation focused on reliability indices and did not assess other sources of validity evidence, such as internal structure, response process, relations to other variables, consequences, or educational impact. These aspects should be examined in future multisite studies.

## Conclusions

By translating complex competencies, such as empathy, professional demeanour, and clinical observation, into observable behaviours with explicit anchors, the checklist/rubric developed in this study may support clearer scoring criteria and more consistent feedback in course-embedded performance assessment. At the same time, scenario-specific variation in inter-rater agreement suggests that some performance elements may benefit from further refinement of behavioural indicators and additional rater training.

Overall, this checklist/rubric provides a practical foundation for strengthening assessment and feedback in intake interview training in mental health occupational therapy. Future research should examine its applicability across additional scenarios and settings, and extend validity evidence by evaluating response processes, educational consequences, and relationships with other performance measures.

## Artificial Intelligence use

The authors used ChatGPT (OpenAI) solely for language suggestions and grammar checks during manuscript preparation. All scientific content, analysis, and interpretation were produced by the authors.

## Additional Files

The additional files for this article can be found as follows:

10.5334/pme.2026.s1 Supplementary Appendix 1. Pilot testing and refinement.

10.5334/pme.2026.s2 Supplementary Figure S1. Bland–Altman plot of inter-rater agreement for total checklist/rubric scores (0–32) in Scenario 1 (n = 20).

10.5334/pme.2026.s3 Supplementary Figure S2. Bland–Altman plot of inter-rater agreement for total checklist/rubric scores (0–32) in Scenario 2 (n = 20).

10.5334/pme.2026.s4 Supplementary Figure S3. Bland–Altman plot of inter-rater agreement for total checklist/rubric scores (0–32) in Scenario 3 (n = 20).

10.5334/pme.2026.s5 Supplementary Table S1. Structure and scoring criteria of the single-station standardised patient assessment (OSCE-format station) for intake interviews in mental health occupational therapy.

10.5334/pme.2026.s6 Supplementary Table S2. Expert panels’ demographic and professional characteristics.

10.5334/pme.2026.s7 Supplementary Table S3. Item-to-construct-and-literature mapping for the checklist/rubric (OSCE-format single-station SP intake interview).

10.5334/pme.2026.s8 Supplementary Table S4. Construct map for intake interview performance.

10.5334/pme.2026.s9 Supplementary Table S5. Descriptive statistics, inter-rater reliability (ICC), and internal consistency (Cronbach’s α) for domain and total scores.

## Data Availability

The datasets generated and analysed during the current study are not publicly available because they contain identifiable educational performance data of students and are subject to institutional privacy regulations, but are available from the corresponding author on reasonable request.
