# Supplementary material for: A Behaviourally Anchored Checklist for Mental Health Occupational Therapy Intake Interviews: Development and Reliability in a Single-Station Standardised Patient Encounter
Source: Perspect Med Educ. 2026 May 7;15(1):410–9. doi: 10.5334/pme.2026 (PMC13155089; doi:10.5334/pme.2026)
Supplement: Supplementary Appendix 1. — Pilot testing and refinement. [file pme-15-1-2026-s1.pdf]

Supplementary Appendix 1. Pilot testing and refinement

Second-year students participated only in the pilot phase and were not included in the main reliability analysis. We piloted the draft checklist/rubric in 2023 with a separate cohort of 20 second-year occupational therapy students from one participating university. The purpose of the pilot was to identify feasibility problems in the station workflow and unclear wording in the behavioural anchors from the learner perspective, rather than to estimate rater agreement or evaluate educational outcomes or psychometric properties.

The pilot station followed the same time structure as the main study, consisting of a 5-minute standardised patient interview and a 5-minute scoring period. In the pilot, the SPs were students who were trained using the same materials and rehearsal procedures as in the main study.

Immediately after the pilot, we collected structured feedback from two sources: (i) assessed students, regarding perceived task feasibility and clarity of prompts and scoring criteria, and (ii) student SPs, regarding feasibility, consistency of role portrayal, and time control. Feedback was obtained through a brief debrief and contemporaneous notes. The research team summarised recurrent issues and prioritised revisions in an expert-panel discussion. Refinements to the behavioural anchors were determined by the expert panel on the basis of these observations and feasibility feedback, rather than on assessed students' opinions about anchor content.

Based on the pilot, we made three types of revisions. First, we clarified the interview prompts by specifying key patient questions to be asked within the station, in order to support consistent information elicitation within the time limit and to improve the observability of related checklist items. Second, we refined the behavioural anchors so that the 0-2 descriptors differed by explicit, observable behaviours and could be applied consistently within the 5-minute scoring period. Third, we adjusted the SP script and training guidance so that delusional responses did not become excessively long and the interaction could reliably conclude within 5 minutes.

Rater consistency was addressed separately through rater training and calibration before the main assessment. The revised version of the checklist/rubric and station procedures was then used in the main study.
