# Supplementary material for: A Behaviourally Anchored Checklist for Mental Health Occupational Therapy Intake Interviews: Development and Reliability in a Single-Station Standardised Patient Encounter
Source: Perspect Med Educ. 2026 May 7;15(1):410–9. doi: 10.5334/pme.2026 (PMC13155089; doi:10.5334/pme.2026)
Supplement: Supplementary Figure S1. — Bland–Altman plot of inter-rater agreement for total checklist/rubric scores (0–32) in Scenario 1 (n = 20). [file pme-15-1-2026-s2.pdf]

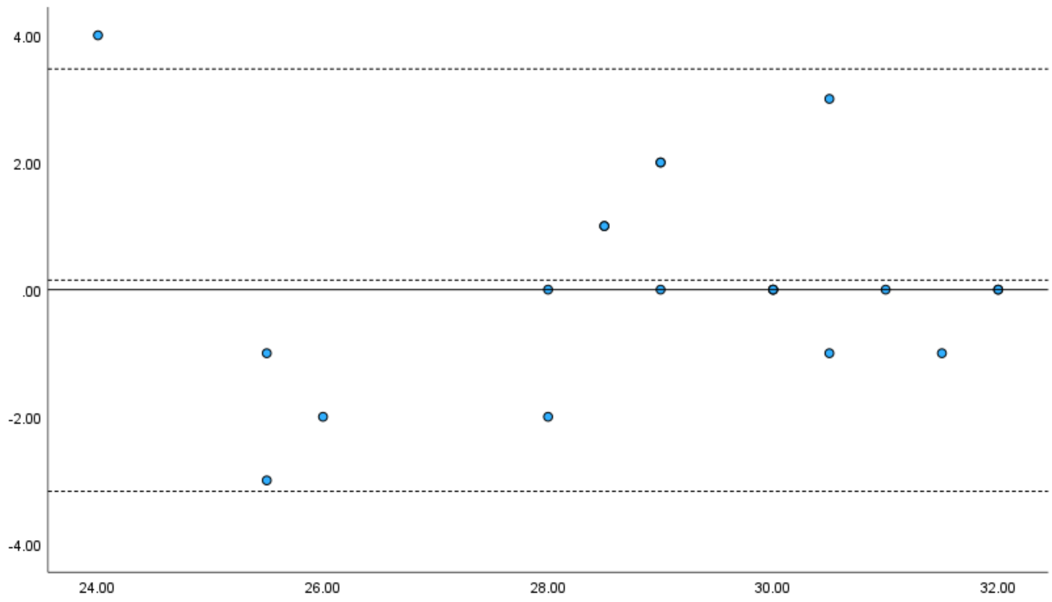

Supplementary Figure S1. Bland-Altman plot of inter-rater agreement for total checklist/rubric scores (0-32) in Scenario 1 (n = 20).

Note. The solid line indicates the mean difference between raters (Rater A − Rater B). The dashed lines indicate the 95% limits of agreement (mean difference ± 1.96 × SD of the differences). X-axis: mean of the two raters' total scores; Y-axis: score difference (Rater A − Rater B). This scenario-specific plot is provided as an exploratory analysis because of the small subgroup size.
