# Supplementary material for: A Behaviourally Anchored Checklist for Mental Health Occupational Therapy Intake Interviews: Development and Reliability in a Single-Station Standardised Patient Encounter
Source: Perspect Med Educ. 2026 May 7;15(1):410–9. doi: 10.5334/pme.2026 (PMC13155089; doi:10.5334/pme.2026)
Supplement: Supplementary Table S1. — Structure and scoring criteria of the single-station standardised patient assessment (OSCE-format station) for intake interviews in mental health occupational therapy. [file pme-15-1-2026-s5.pdf]

Supplementary Table S1. Structure and scoring criteria of the single-station standardised patient assessment (OSCE-format station) for intake interviews in mental health occupational therapy

No.	Category	Item	Description	Scoring Criteria (0–2 pts)
1	Attitude	Greeting and self-introduction	Appropriate attire, clear greeting, and self-introduction	2 = All 3 fulfilled<br>1 = 1 missed<br>0 = ≥2 missed
2	Attitude	Patient identification	Verification using at least two identifiers (e.g., name, DOB, and ID)	2 = ≥2 identifiers<br>1 = 1 identifier<br>0 = None
3	Attitude	Explanation of the purpose and duration	Explains the purpose, content, and approximate duration of the interview	2 = All 3 explained<br>1 = 1 missed<br>0 = ≥2 missed
4	Attitude	Obtaining consent	Informs the patient about the interview and note-taking, and obtains consent	2 = Both items explained<br>1 = 1 only<br>0 = None
5	Skills	Seating and distance	Guides patients to their seats with appropriate distance and 90° angle; the patient sits first	2 = All 3 fulfilled<br>1 = 1 missed<br>0 = ≥2 missed
6	Skills	Verbal clarity	Maintains appropriate language, pace, and volume throughout	2 = All 3 maintained<br>1 = 1 missed<br>0 = ≥2 missed
7	Skills	Non-verbal behaviour	Maintains proper facial expressions, gestures, and posture	2 = All 3 maintained<br>1 = 1 missed<br>0 = ≥2 missed
8	Skills	Active listening	Uses appropriate listening techniques (e.g., nodding and echoing)	2 = Appropriate use throughout<br>1 = 1–2 inappropriate uses<br>0 = ≥3 inappropriate uses
9	Skills	Open-ended questioning	Uses open-ended questions to identify	2 = Both identified<br>1 = 1 only

			main complaints and needs	0 = None
10	Skills	Empathy and neutrality	Shows empathy without denying or affirming the patient’s experiences	2 = Both achieved<br>1 = 1 only<br>0 = None
11	Skills	Summarizing and confirming	Summarizes and checks the accuracy of the information with the patient	2 = Both done<br>1 = 1 only<br>0 = None
12	Skills	Closure	Checks for additional questions, makes next appointment, and expresses gratitude	2 = All 3 done<br>1 = 1 missed<br>0 = ≥2 missed
13	Skills	Professional response	Responds appropriately as an OT when facing questions/comments	2 = Appropriate throughout<br>1 = 1 inappropriate response<br>0 = ≥2 inappropriate
14	Evaluation	Observation reporting (appearance, expression, and posture)	Accurately reports observed physical cues during the interview	2 = All 3 reported<br>1 = 1 missed<br>0 = ≥2 missed
15	Evaluation	Speech and thought characteristics	Reports speaking style and mental symptoms (e.g., psychotic features)	2 = Both domains reported<br>1 = 1 only<br>0 = None
16	Evaluation	Content summary	Accurately summarizes and reports the patient’s words	2 = Both accurate<br>1 = 1 only<br>0 = None

Scoring Criteria: The checklist/rubric comprises three domains: attitude (0–8 points), interview skills (0–18 points), and evaluation (0–6 points), with a total possible score of 0–32 points. Each item is rated on a 3-point scale (0 = not demonstrated, 1 = partially demonstrated, 2 = fully demonstrated) based on observable behaviours relevant to psychiatric intake interviews.
