# Supplementary material for: A Behaviourally Anchored Checklist for Mental Health Occupational Therapy Intake Interviews: Development and Reliability in a Single-Station Standardised Patient Encounter
Source: Perspect Med Educ. 2026 May 7;15(1):410–9. doi: 10.5334/pme.2026 (PMC13155089; doi:10.5334/pme.2026)
Supplement: Supplementary Table S2. — Expert panels’ demographic and professional characteristics. [file pme-15-1-2026-s6.pdf]

Supplementary Table S2. Expert panels' demographic and professional characteristics

Characteristic	Category	n
Sex	Male	5
Age group (years)	30–39	3
	40–49	2
Primary role	Occupational therapy educator (mental health)	2
	Clinical occupational therapist (mental health)	3
Clinical experience (years)	10–19	3
	20–29	2
Practice setting	University	2
	Hospital or psychiatric daycare	2
	Community-based rehabilitation	1

Abbreviations: OT, occupational therapist; OSCE, Objective Structured Clinical Examination.
To preserve anonymity, institution names and geographic locations are not reported.
