# Supplementary material for: A Behaviourally Anchored Checklist for Mental Health Occupational Therapy Intake Interviews: Development and Reliability in a Single-Station Standardised Patient Encounter
Source: Perspect Med Educ. 2026 May 7;15(1):410–9. doi: 10.5334/pme.2026 (PMC13155089; doi:10.5334/pme.2026)
Supplement: Supplementary Table S3. — Item-to-construct-and-literature mapping for the checklist/rubric (OSCE-format single-station SP intake interview). [file pme-15-1-2026-s7.pdf]

Supplementary Table S3. Item-to-construct-and-literature mapping for the checklist/rubric (OSCE-format single-station SP intake interview)

Domain	Item No.	Item (exact label)	Target construct (short)	Key sources	Design link (how prior literature informed this item in this context)
Attitude	1	Greeting and self-introduction	Professional opening; rapport	[6, 7, 15]	Therapeutic communication and professional demeanour were translated into observable opening behaviours.
Attitude	2	Patient identification	Safety/identity verification	[6, 7, 15]	Professional conduct standards were operationalised as explicit verification behaviours feasible in a brief station.
Attitude	3	Explanation of the purpose and duration	Transparency; agenda setting	[6, 7, 15]	Patient-centred communication was operationalised as clear purpose/content/time explanation to support engagement.
Attitude	4	Obtaining consent	Informed consent in encounter	[6, 7, 15]	Ethical/professional stance was anchored as explicit consent for interview and note-taking.
Interview skills	5	Seating and distance	Interactional setup; engagement	[19]	Station-specific evidence/practice on seating, distance, and positioning informed a scorable behavioural sequence.
Interview skills	6	Verbal clarity	Clarity of speech; accessibility	[6, 7, 15]	Communication quality (language, pace, volume) was translated into three observable criteria for real-time scoring.
Interview	7	Non-verbal	Nonverbal rapport	[6, 7, 15]	Nonverbal aspects of

skills		behaviour	behaviours		therapeutic communication were operationalised as facial expression, gesture, and posture.
Interview skills	8	Active listening	Listening micro-skills	[6,7,15]	Listening behaviours (e.g., nodding/echoing) were anchored as observable techniques used during information gathering.
Interview skills	9	Open-ended questioning	Eliciting concerns/needs	[6,7,15]	Interview communication literature informed the use of open-ended questions to elicit main complaints and needs.
Interview skills	10	Empathy and neutrality	Empathic stance without validation/denial	[6,7,15]	Empathy and neutrality were defined as a dual requirement and anchored as behaviours appropriate for psychiatric intake.
Interview skills	11	Summarising and confirming	Shared understanding/check-back	[6,7,15]	Summarising and checking accuracy were anchored as explicit "summary + confirmation" behaviours.
Interview skills	12	Closure	Structured ending; next steps	[6,7,15]	Patient-centred closing behaviours were operationalised as questions, next appointment, and gratitude within time limits.
Interview skills	13	Professional response	OT-appropriate professional responding	[6,7,15]	Professional role communication was anchored as appropriate OT responses to patient questions/comments in the station.
Evaluation	14	Observation reporting	Mental status observation: visible cues	[17]	Psychiatric interview literature informed

		(appearance, expression, and posture)			which observable cues should be noted and reported after the encounter.
Evaluation	15	Speech and thought characteristics	Mental status observation: speech/thought	[17]	Initial assessment priorities (speech/thought content and psychotic features) were translated into scorable reporting elements.
Evaluation	16	Content summary	Brief synthesis of patient narrative	[17]	Brief synthesis/reporting of the patient's words was anchored as accurate summarisation under time constraints.

Note: "Key sources" indicate the literature streams used to justify item inclusion and/or anchoring. Full citations are provided in the main reference list. In addition, the behavioural anchoring approach and real-time station-based scoring design applied to all items were informed by OSCE/simulation-based assessment literature [12,13,16].
