# Supplementary material for: A Behaviourally Anchored Checklist for Mental Health Occupational Therapy Intake Interviews: Development and Reliability in a Single-Station Standardised Patient Encounter
Source: Perspect Med Educ. 2026 May 7;15(1):410–9. doi: 10.5334/pme.2026 (PMC13155089; doi:10.5334/pme.2026)
Supplement: Supplementary Table S4. — Construct map for intake interview performance. [file pme-15-1-2026-s8.pdf]

Supplementary Table S4. Construct map for intake interview performance

Construct map element	Operational definition (1 sentence)	Examples of observable indicators in the station	Instrument domain	Key literature stream
Professional/relational stance	Learner maintains respectful, empathic, and professional engagement throughout the encounter.	Greeting/self-introduction; consent; empathic but neutral responses; appropriate nonverbal behaviour.	Attitude / Interview skills	OT therapeutic communication [6,7,15]
Interview-process behaviours	Learner structures the interaction to elicit information efficiently and coherently within time limits.	Open-ended questioning; active listening; summarising/check-back; closure/next steps.	Interview skills	OT therapeutic communication [6,7,15] + OSCE/simulation assessment design [12,13,16]
Observation and brief synthesis/reporting	Learner observes clinically relevant presentation cues and produces a concise synthesis/report.	Reports appearance/posture/expression; speech/thought characteristics; content summary.	Evaluation	Psychiatric interview literature [17]
