# Supplementary material for: A Behaviourally Anchored Checklist for Mental Health Occupational Therapy Intake Interviews: Development and Reliability in a Single-Station Standardised Patient Encounter
Source: Perspect Med Educ. 2026 May 7;15(1):410–9. doi: 10.5334/pme.2026 (PMC13155089; doi:10.5334/pme.2026)
Supplement: Supplementary Table S5. — Descriptive statistics, inter-rater reliability (ICC), and internal consistency (Cronbach’s α) for domain and total scores. [file pme-15-1-2026-s9.pdf]

Supplementary Table S5. Descriptive statistics, inter-rater reliability (ICC), and internal consistency (Cronbach's α) for domain and total scores

Domain	Rater	Mean (SD)	Median (Range)	SD%max	ICC (95% CI)	Cronbach's α
Attitude (0–8)	A	7.70(0.56)	8(6–8)	7.0%	0.765 (0.638–0.852)	0.885
	B	7.69(0.56)	8(5–8)	7.0%		0.887
Interview skills (0–18)	A	16.46(1.31)	17(13–18)	7.3%	0.670 (0.506–0.788)	0.853
	B	16.57(1.35)	17(11–18)	7.5%		0.855
Evaluation (0–6)	A	5.16 (1.45)	6(0–6)	24.2%	0.900 (0.839–0.938)	0.850
	B	4.89 (1.51)	6(0–6)	25.2%		0.843
Total (0–32)	A	29.33(2.33)	30(24–32)	7.3%	0.854 (0.768–0.909)	0.816
	B	29.15(2.43)	29(22–32)	7.6%		0.817

Note. Values are reported as median (range) and mean (SD). Range indicates the minimum and maximum observed scores. SD%max = SD divided by the domain maximum score × 100.
